# Screening for Neuraminidase Inhibitory Activity in Traditional Chinese Medicines Used to Treat Influenza

**DOI:** 10.3390/molecules21091138

**Published:** 2016-08-27

**Authors:** Xian-Ying Yang, Ai-lin Liu, Shu-jing Liu, Xiao-wei Xu, Lin-Fang Huang

**Affiliations:** 1 Institute of Medicinal Plant Development, Chinese Academy of Medical Sciences & Peking Union Medical College, Beijing 100193, China; 18787292050@139.com; 2 College of Pharmacy and Chemistry, Dali University, Yunnan 671000, China; 3 Institute of Materia Medica, Chinese Academy of Medical Sciences & Peking Union Medical College, Beijing 100050, China; liuailin@imm.ac.cn; 4 Department of Pathology and Laboratory Medicine, Perelman School of Medicine, University of Pennsylvania, Philadelphia, PA 19104, USA; shujing@mail.med.upenn.edu (S.L.); xug@mail.med.upenn.edu (X.X.)

**Keywords:** neuraminidase inhibition, screening, traditional Chinese medicine

## Abstract

Objective: To screen for influenza virus neuraminidase inhibition and to provide a reference for the clinical treatment of influenza using traditional Chinese medicines (TCM). In this study, 421 crude extracts (solubilized with petroleum ether, ethanol, ethyl acetate, and aqueous solvents) were obtained from 113 TCM. The medicine extracts were then reacted with oseltamivir, using 2’-(4-methylumbelliferyl)-α-D-*N*-acetylneuraminic acid (MUNANA) as the substrate, to determine influenza virus neuraminidase activity using a standard fluorimetric assay. It was found that Chinese medicine extracts from *Pyrola calliantha*, *Cynanchum wilfordii*, *Balanophora involucrata* and *Paeonia delavayi* significantly inhibited neuraminidase activity at a concentration of 40 μg/mL. Dose-dependent inhibitory assays also revealed significant inhibition. The IC_50_ range of the TCM extracts for influenza virus neuraminidase was approximately 12.66–34.85 μg/mL, respectively. Some Chinese medicines have clear anti-influenza viral effects that may play an important role in the treatment of influenza through the inhibition of viral neuraminidase. The results of this study demonstrated that plant medicines can serve as a useful source of neuraminidase (NA) inhibitors and further investigation into the pharmacologic activities of these extracts is warranted.

## 1. Introduction

Influenza (flu) is an infectious disease that seriously affects human life and health [1,2]. According to the World Health Organization (WHO) statistics, influenza annually causes an estimated 250,000–500,000 deaths and approximately three to five million cases of severe illness worldwide. Influenza poses a range of serious threats to public health by inducing substantial economic losses and social problems throughout the world [3,4].

Influenza A viruses, including the H5N1, H3N2 and H1N1 subtypes, pose a potential pandemic threat to public health [1]. According to World Health Organization (WHO) statistics, as of January 2014, there have been a total of 650 confirmed human cases of H5N1 virus, with 386 deaths (59% mortality rate) in 15 countries since 2003 [5].

At present, there are two available classes of anti-influenza viral drugs: NA inhibitors (oseltamivir, zanamivir, peramivir and laninamivir) and M2 ion channel inhibitors (amantadine and rimantadine) [6]. NA inhibitors were developed because of the genetic stability of the influenza virus active NA enzymatic center [7]. NA is an influenza virus surface glycoprotein that is recognized as an attractive target for the development of antiviral drugs [8,9]. Currently, neuraminidase inhibitors (NAIs) are in wide use for the treatment of influenza [10]. However, the efficacy of these drugs has declined due to viral mutations conferring resistance to some NAIs [11]. Because of this challenge, many researchers are now focused on the development of new anti-influenza treatments or combination therapies to enhance the efficacy of anti-influenza drugs [12,13].

Although synthetic NAIs, such as seltamivir and zanamivir, have been designed to halt viral replication, adverse side effects, such as nausea, vomiting, diarrhea, abdominal pain, have been observed [14,15]. Hence, naturally existing NAIs have attracted considerable interest for treating influenza [16,17]. Additionally, compound indigowoad root granules and ginseng polysaccharides have been recognized as antiviral agents with activity against the influenza virus [9]. Many Chinese traditional patent medicines, such as Shuanghuanglian oral liquid, Qingkailing oral liquid, Qingre Jiedu oral liquid and Reduning injection, have also displayed relatively high NA inhibitory activities. 

In this study, 421 crude extracts (solubilized with petroleum ether, ethanol, ethyl acetate, and aqueous solvents) were obtained from 113 traditional Chinese medicines. Some plant medicines have clear anti-influenza viral effects. The results of this study will provide important information for the isolation of active constituents and for the clinical use of TCM for treating and preventing influenza.

## 2. Materials and Methods 

### 2.1. Plant Materials 

All TCM were collected from Yun Nan and Si Chuan provinces by Professor Linfang Huang. The identities of all samples were authenticated by Professor Yulin Li. The selected specimens were deposited in the herbarium of the Institute of Medicinal Plant Development, Chinese Academy of Medical Sciences. 

### 2.2. Chemicals

Chemicals used included 2’-(4-methylumbelliferyl)-α-D-*N*-acetylneuraminic acid (MUNANA, Sigma, St. Louis, MO, USA), MES, (Sigma, St. Louis, MO, USA), CaCl_2_, NaOH, absolute ethyl alcohol (pure analytical grade), and other chemicals, all of which were of extra pure analytical grade.

### 2.3. Plant Extraction 

**Figure 1:**
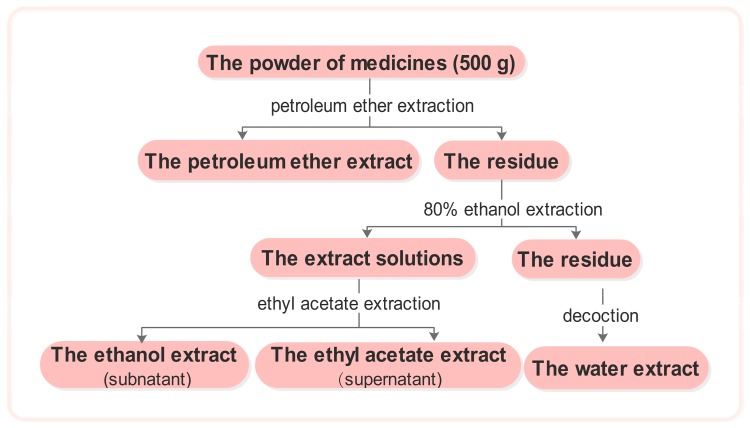
The extract flow chart of the 113 traditional Chinese medicines.

The medicinal plant material was crushed into coarse powder. Five hundred grams of powder was soaked in petroleum ether for 24 h, after which a percolation extraction was performed. The filter was retrieved and the petroleum ether was evaporated. The residue was washed with 80% ethanol and subjected twice to reflux extraction with triple the volume of 80% ethanol. The extract solutions were then combined and ethanol was reclaimed at reduced pressures until no alcohol was detected. Extraction was then performed twice with an equal volume of ethyl acetate. The upper solution was then extracted and concentrated to obtain the ethyl acetate extract, whereas the lower solution was concentrated to dryness to yield the ethanol extract. The residue was evaporated to dryness and was then extracted twice with an amount of water equal to triple the mass of the materials. The aqueous extract solutions were combined and concentrated to dryness, and the water extract was then obtained (Figure 1). 

### 2.4. Neuraminidase Inhibition Assay 

**Figure 2:**
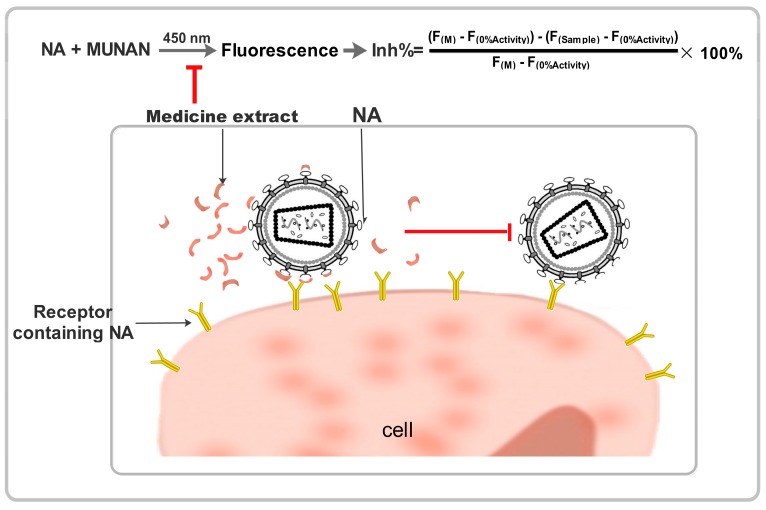
Schematic diagrams showing the neuraminidase inhibiting effect of Chinese medicine.

The substrate 2’-(4-methylumbelliferyl)-α-D-*N*-acetylneuraminic acid (MUNANA) was combined with oseltamivir or traditional Chinese medicine extracts to examine influenza virus NA activity using a standard fluorimetric assay. In this assay, the substrate and NA reacted to yield a fluorescent product that could be quantified [6,18] (Figure 2).

The reaction mixture containing test extract compounds and either NA enzyme or a viral suspension in 33 mM MES buffer and 4 mM calcium chloride (pH 6.5) was incubated for 40 min at 37 °C. After incubation, the reaction was terminated by adding 34 mM NaOH. Fluorescence was quantified at an excitation wavelength of 360 nm and an emission wavelength of 450 nm. The 50% inhibitory concentration (IC_50_) was defined as the concentration of NA inhibitor necessary to reduce NA activity by 50% relative to a reaction mixture containing virus but no inhibitor. The data were expressed as the mean of six independent experiments.

## 3. Results and Discussion

The inhibitory activities on NA for the TCM species examined were evaluated and the percentage inhibitions are shown in Table 1.

Four extracts using petroleum ether, ethyl acetate, ethanol and aqueous extracts were prepared from each of the 113 dried medicines. The TCM extracts were analyzed for NA inhibitory activity. Twenty-six of the extracts (from *Citrus reticulata* Blanco, *Angelica pubescens and Radix Anemones* Rivularis species) were found to promote NA activity, whereas 395 extracts showed different degrees of NA inhibitory activity. Twenty-six extracts were found to inhibit NA by greater than 50%, including the 11 ethanol extracts of *Curcuma longa* *L*., *Rhus chinensis* *Mill*., *Fagopyrum dibotrys* and *Fagopyrum dibotrys species.* Furthermore, the 12 ethyl acetate extracts of *Balanophora involucrata*, *Balanophora involucrata*, *Paeonia delavayi* *Franch*, and *Cynanchum wilfordii* (Maxim.) *Hemsl*.; the three petroleum ether extracts of *Carthamus tinctorius* *L*., *Fagopyrum dibotrys*, *Polygonum aubertii Henry*; and the three aqueous extracts of *Cynanchum wilfordii, Paeonia delavayi* *Franch* and *Rhus chinensis* *Mill*. exhibited significant NA inhibition at 40 μg/mL. 

The dose-dependent NA inhibitory activities of 10 medicines that exhibited the most NA inhibition were studied further. The IC_50_ inhibition values are presented in Table 2. Among these 10 TCM, the most potent NA inhibition was exhibited by the ethyl acetate extract of *Paeonia delavayi* Franch (IC_50_ = 12.66 μg/mL).

Influenza is a serious threat to human health. Thus, there is an urgent need to develop anti-influenza drugs. Some herbal medicines are used as a treatment for influenza. Traditional Chinese medicines may have an important role in the research and development of new drugs for influenza treatment. Screening for bioactive compounds from medicinal plants is an important strategy. NAIs from TCM are important resources for potential therapeutic agents directed against influenza. 

This paper evaluated the in vitro activity of commonly used TCM against influenza virus neuraminidase. Here, we screened novel NAI extracted from 113 medicines using a fluorimetric assay. These results suggest that *Rhus chinensis* and *Paeonia delavayi* offer great potential for the treatment of influenza. Most of the ethyl acetate extracts showed strong NA inhibitory activities. This is the first time that medicine extracts have been tested on a large scale for their ability to inhibit NA. In addition, the 10 TCM that exhibited the most NAI in this study have not been traditionally used to treat influenza. Among these 10 medicine extracts, the *Paeonia delavayi* ethyl acetate extracts were the most potent in the NAI assays. 

According to the Chinese pharmacopoeia (2015, [19]) and other references, all 10 TCM have the effects of heat-clearing and detoxification. It is believed that heat-clearing and detoxification are connected with eliminating the virus, while the support of healthy energy is concerned with enhancing immunity. Influenza is treated by drugs to relieve the ‘exterior syndrome’, and heat-clearing drugs are used as antibiotics [15]. 

Interestingly, some medicines (*Isatis indigotica, Forsythia suspensa, Lonicera japonica* and *Scutellaria baicalensis*) that have traditionally been prescribed to treat influenza were found to have low anti-NA activity at 40 μg/mL. The inhibition by *Isatis indigotica* was less than 5%. The data indicated that the anti-influenza effect of this medicine is not influenced by the effect of inhibiting NA.

## 4. Conclusions

The results of this study indicate that many plant medicines offer great potential for the treatment of influenza. The full therapeutic range of traditional Chinese medicines has been relatively unexplored. The results of this report warrant further investigation of TCM extracts for potential therapeutic agents to use in the treatment of influenza. The anti-influenza activity of NAIs has been well established by numerous in vitro and in vivo studies. However, there is scarcity in the volume of the cell experiments and in vivo studies undertaken to explore these TCM potentials for anti-influenza activity. In the future, we will make an effort to identify the bioactive components of the extracts and explore the antiviral activity of these compounds with in vivo and in vitro experiments.

## Figures and Tables

**Table 1:** Effects of selected traditional Chinese medicines (extracts) on inhibition of NA.

No.	Medicinally used Parts Herbs name	Medicinally Used Parts	Percentage Inhibition (%)			
			P	E	EA	W
1	*Rubia yunnanensis*	Root	5.65	7.45	10.22	0.11
2	*Boschniakia himalaica*	Root tuber	11.85	21.32	11.17	8.18
3	*Astragalus membranaceus*	Root	11.36	12.52	15.23	13.93
4	*Achyranthes aspera*	Whole Plant	23.86	4.45	18.42	43.67
5	*Carthamus tinctorius*	Flower	51.33	33.97	17.16	23.60
6	*Rodgersia pinnata*	Rhizome	22.59	15.34	16.71	13.60
7	*Morus alba*	Root bark	15.72	8.11	23.76	17.25
8	*Cibotium barometz*	Rhizome	19.38	33.72	19.12	19.12
9	*Lonicera japonica*	Flower	10.89	49.53	-	49.53
10	*Gossampinus malabarica*	Flower	−7.88	25.47	-	26.06
11	*Gastrodia elata*	Tuber	-	18.49	-	28.33
12	*Aconitum brachypodum*	Root	-	28.33	23.79	19.37
13	*Pyrola calliantha*	Whole Plant	3.76	70.49	79.10	27.83
14	*Potentilla griffithii*	Root	21.43	29.19	19.21	27.24
15	*Scutellaria baicalensis*	Root	9.74	58.38	30.83	29.46
16	*Geranium strictipes*	Root	14.70	50.01	84.69	59.91
17	*Sinomenium acutum.*	Stem	−3.96	25.88	20.85	18.69
18	*Choerospondias axillaris*	Fruit	−3.24	8.04	12.38	10.95
19	*Aster tataricus*	Rhizome	−24.49	15.92	23.25	−3.80
20	*Citrus reticulata*	Seed	−28.91	2.07	−12.07	−1.01
21	*Balanophora involucrata*	Whole Plant	4.83	63.29	63.72	42.74
22	*Rubus delavayi*	Whole Plant	8.19	42.30	57.38	38.97
23	*Bidens bipinnata*	Whole Plant	−2.39	16.09	28.01	19.65
24	*Saururus chinensis*	Whole Plant	20.45	30.31	26.45	20.70
25	*Erigeron breviscapus*	Whole Plant	29.04	25.51	24.92	17.35
26	*Laggera pterodonta*	Whole Plant	23.46	40.31	36.92	34.77
27	*Cynanchum otophyllum*	Root	26.88	29.17	1.71	20.23
28	*Marsdenia tenacissima*	Rattan	15.41	26.10	39.65	30.58
29	*Platycladus orientalis*	leaf	28.98	53.49	37.77	35.36
30	*Euphorbia hirta*	Whole Plant	25.88	48.97	61.03	29.95
31	*Paeonia delavayi*	Root	25.50	78.83	91.85	50.06
32	*Hedyotis diffusa*	Whole Plant	33.40	33.12	28.40	24.47
33	*Juglans regia*	Seed	17.81	52.46	40.47	34.59
34	*Forsythia suspensa*	Fruit	20.95	57.66	29.89	27.63
35	*Terminalia chebula*	Fruit	24.55	41.59	33.40	36.07
36	*Triplostegia glandulifera*	Root	10.78	18.28	29.09	19.65
37	*Sophora japonica*	Fruit	16.87	32.55	45.39	32.70
38	*Anemone rivularis*	leaf	0.65	−6.81	−25.93	8.50
39	*Angelica pubescens*	Root	−17.19	−28.15	−76.53	−0.24
40	*Amomum tsaoko*	Fruit	−7.99	0.37	3.43	−3.22
41	*Areca catechu*	Seed	1.26	46.00	-	27.99
42	*Plantago depressssa*	Seed	-	12.27	4.26	5.10
43	*Isatis indigotica*	Root	-	4.19	-	0.59
44	*Alisma orientalis*	Tuber	-	−2.95	3.16	5.88
45	*Verbena officinalis*	Whole Plant	12.13	27.97	53.30	12.73
46	*Acalypha australis*	Whole Plant	15.37	22.54	29.87	8.26
47	*Campylotropis trigonoclada*	Whole Plant	7.94	20.47	29.92	17.01
48	*Triplostegia glandulifera*	Rhizome	14.49	20.09	22.04	20.00
49	*Fagopyrum dibotrys*	Root tuber	54.11	**70.41**	-	17.50
50	*Gonostegia hirta*	Root	14.84	19.73	27.96	17.58
51	*Elsholtzia bodinieri*	Whole Plant	21.68	42.61	38.18	20.71
52	*Reineckia carnea*	Whole Plant	17.73	3.89	10.98	16.83
53	*Euphorbia sessiliflora*	Whole Plant	19.29	16.57	24.21	21.24
54	*Pyrrosia petiolosa*	leaf	23.98	26.45	35.21	13.88
55	*Euphorbia lathyris*	Fruit	5.29	13.53	28.39	14.87
56	*Boenninghausenia sessilicarpa*	Whole Plant	0.21	21.85	−38.94	15.56
57	*Angelica sinensis*	Seed	−6.14	4.18	-	10.94
58	*Hydnocarpus anthelminthicus*	Fruit	11.01	11.44	33.46	14.54
59	*Psoralea corylifolia*	Fruit	14.72	14.90	38.37	0.83
60	*Mahonia bealei*	Root	−4.42	5.57	-	−2.46
61	*Inula linariifolia*	Whole Plant	6.78	9.69	-	4.33
62	*Acorus calamus*	Rhizome	4.40	2.33	−3.89	-
63	*Rosa laevigata*	Root	-	0.63	11.59	6.79
64	*Cistanche deserticola*	Stem	11.88	20.96	-	-
65	*Phyllanthus emblica*	Fruit	11.30	29.50	45.21	-
66	*Stellera chamaejasme*	Root	7.88	6.13	-	2.75
67	*Piper longum*	Whole Plant	14.15	26.01	22.89	22.62
68	*Geum aleppicum*	Whole Plant	24.05	37.39	36.92	17.78
69	*Ailanthus altissima*	Whole Plant	20.74	4.01	24.14	20.83
70	*Epimedium brevicornu*	leaf	34.99	30.13	32.70	26.97
71	*Bombyx mori*	Excreta	25.31	21.65	13.77	12.88
72	*Paeonia lactiflora*	Root	-	34.96	59.63	29.28
73	*Dioscorea opposita*	Root	29.86	28.48	33.41	28.87
74	*Crotalaria ferruginea* *.*	Whole Plant	34.63	31.24	33.04	24.32
75	*Inula japonica* *.*	Flower	26.06	47.10	-	40.29
76	*Rhizoma Scirpi*	Root tuber	33.81	30.22	29.77	-
77	*Tussilago farfara*	Flower	21.03	27.57	-	24.38
78	*Polygonum multiflorum*	Root tuber	19.68	**75.13**	**78.72**	-
79	*Cistanche deserticola*	Succulent stem	-	25.92	25.18	34.42
80	*Pyrrosia petiolosa*	leaf	29.08	17.04	37.20	10.67
81	*Paederia scandens*	Whole Plant	17.21	23.37	-	25.82
82	*Entada phaseoloides*	Seed	23.61	25.13	-	23.46
83	*Cyperus rotundus*	Rhizome	-	23.44	23.00	14.28
84	*Rosmarinus officinalis*	leaf	15.52	18.94	18.94	33.39
85	*Siphonostegia chinensis*	Whole Plant	12.75	16.31	16.31	12.37
86	*Rhus chinensis*	Insect gall	23.19	**72.08**	**84.08**	**66.53**
87	*Caesalpinia sappan*	Duramen	-	18.65	16.88	12.56
88	*Corydalis pallida*	Root	25.19	12.55	26.79	10.31
89	*Uncaria macrophylla*	leaf	1.87	30.63	33.35	14.66
90	*Lycium chinense*	Velamen	-	5.22	11.47	7.85
91	*Codonopsis pilosula*	Root	12.06	15.65	8.74	7.77
92	*Semen Persicae*	Seed	8.34	7.51	-2.84	3.59
93	*Lonicera japonica*	Flower	15.15	17.76	17.76	13.61
94	*Polygonum auberti* *i*	Root	**86.12**	11.05	12.59	27.53
95	*Cynomorium songaricum*	Succulent stem	−2.09	7.51	34.59	6.64
96	*Cnidium monnieri*	Fruit	2.45	2.10	−64.17	3.96
97	*Eucommia ulmoides*	bark	3.59	6.48	15.46	4.02
98	*Equisetum arvense*	Whole Plant	7.13	12.56	23.32	8.53
99	*Portulaca oleracea*	Whole Plant	11.74	14.18	16.18	6.51
100	*Equisetum hiemale*	Acrial part	13.74	23.70	15.55	11.56
101	*Clematis manshurica*	Rhizome	11.90	13.35	19.36	9.76
102	*Notopterygium incisun*	Rhizome	10.24	−7.62	5.29	20.01
103	*Dioscorea nipponica*	Rhizome	13.02	13.35	21.27	12.48
104	*Anemarrhena asphodeloides* *．*	Rhizome	15.76	21.47	42.45	14.12
105	*Dictamnus dasycarpus*	root bark	7.87	16.32	11.18	15.52
106	*Panax ginseng*	Rhizome, Root	13.32	18.69	24.95	11.20
107	*Salvia miltiorrhiza*	leaf	23.03	26.77	**54.48**	28.53
108	*Ligusttcum chuanxiong*	Rhizome	12.64	17.72	22.81	15.11
109	*Leonurus japonicus*	Acrial part	14.38	20.98	24.48	13.80
110	*Xanthium sibiricum*	Seed	11.72	26.70	36.24	18.74
111	*Cannabis sativa*	kernel	15.40	13.68	34.42	8.16
112	*Ginkgo biloba*	leaf	13.51	22.16	38.85	22.55
113	*Curcuma longa*	Rhizome	18.09	53.42	**77.11**	14.30

P—The petroleum ether extract; E—The ethanol extract; EA—The ethyl acetate extract; W—The aqueous extract.

**Table 2:** IC_50_ values for NA inhibitors of the petroleum ether, ethanol, ethyl acetate, and aqueous extracts from 10 traditional Chinese medicines.

No.	Herbs Name	IC_50_ Value (μg/mL)			
		P	E	EA	W
1	*Pyrola calliantha*	-	-	34.4 ± 1.18	-
2	*Cynanchum wilfordii*	-	-	27.84 ± 1.72	
3	*Balanophora involucrata*	-	-	34.85 ± 0.95	-
4	*Paeonia delavayi.*	-	33.64 ± 1.82	12.66 ± 0.87	-
5	*Fagopyrum dibotrys*	-	31.92 ± 1.03	-	-
6	*Polygonum multiflorum*	-	31.92 ± 0.84	28.77 ± 1.68	-
7	*Rhus chinensis*	-	28.24 ± 1.01	19.26 ±1.52	33.54 ± 0.85
8	*Polygonum aubertii*	30.94 ± 1.35	-	-	-
9	*Salvia miltiorrhiza*	-	-	27.33 ± 1.34	-
10	*Curcuma longa*	-	30.26 ± 1.37	25.38 ± 1.51	-

P—The petroleum ether extract; E—The ethanol extract; EA—The ethyl acetate extract; W—The aqueous extract; Values are expressed as mean ±SD (*n* = 3).
